# Keystone Microbiomes Revealed by 14 Years of Field Restoration of the Degraded Agricultural Soil Under Distinct Vegetation Scenarios

**DOI:** 10.3389/fmicb.2020.01915

**Published:** 2020-08-18

**Authors:** Zhiming Zhang, Xiaozeng Han, Jun Yan, Wenxiu Zou, Entao Wang, Xinchun Lu, Xu Chen

**Affiliations:** ^1^National Observation Station of Hailun Agro-Ecology System, Key Laboratory of Mollisols Agroecology, Northeast Institute of Geography and Agroecology, Chinese Academy of Sciences, Harbin, China; ^2^Departamento de Microbiología, Escuela Nacional de Ciencias Biológicas, Instituto Politécnico Nacional, Mexico City, Mexico

**Keywords:** ecological restoration, microbial-community composition, metabolism of carbon sources, soil nutrient contents, black soil

## Abstract

Agricultural intensification accelerates the degradation of cropland, and restoration has been managed by changing its vegetation. However, the keystone microbiome that drives the decomposition of plant-associated organic matter in the restoration is poorly understood. In this study, we established a 14-year field restoration experiment on a degraded cropland with four treatments: (1) bare land soil without biomass input (BL), (2) maize cropland (CL) without fertilization and biomass input, (3) natural grassland (GL), and (4) alfalfa cropland (AL) with biomass left in the fields. The activity of total soil microbiome was assessed by community-level physiological profiling (CLPP) with Biolog EcoPlates analysis, and keystone microbiome was identified as phylotypes showing statistically significant increase in the restored soils (GL and AL) relative to the degraded BL soil. The results showed that GL and AL treatments improved soil fertility as indicated by significant increase in soil organic carbon, total nitrogen, and available phosphorus when compared to BL treatment. The significant difference was not observed between CL and BL treatments except for pH and available phosphorus, indicating that the input and microbial decomposition of plant-associated organic matter were the key for restoration of soil fertility. Similar results were obtained for soil microbial activities of carbon utilization efficiency via CLPP analysis, and real-time quantitative polymerase chain reaction of 16S rRNA genes further revealed significantly higher abundance of total soil microbial community in GL and AL soils than in BL and CL. High-throughput sequencing of total 16S rRNA genes revealed the Bacteroidetes as the only keystone taxa at phylum level, and 106 and 120 genera were keystone phylotypes. Compared with BL and CL, the genera that increase significantly in GL and AL are called keystone genera of ecological restoration. The dominant keystone genera included *Reyranella*, *Mesorhizobium*, *Devosia*, *Haliangium*, *Nocardioides*, and *Pseudonocardia*. Significantly higher abundance of *Bacillus* genus in BL soil implied it might serve as an indicator of agricultural land degradation. Statistical analysis showed that soil organic carbon and pH were significantly correlated with the input of plant-associated organic matters and dynamic changes of keystone taxa. These results suggest that the vegetation of natural grass (GL) and alfalfa plant (AL) and subsequent decomposition of plant-associated materials could serve as effective strategies for restoration of the degraded cropland by stimulating the keystone microbiomes and improving their physiological metabolisms of carbon utilization efficiency.

## Introduction

Soil degradation is of great concern in every agricultural region of the world because of its long-term negative effects on soil productivity (Bindraban et al., 2012; Gong et al., 2013). Conventional agriculture managements, such as intensive monoculture, tilling, and fertilization, can degrade soil and fragment habitats, which can in turn affect the health of agricultural ecosystems and threaten the survival of humans (Foley et al., 2005). Revegetation is a widely accepted restoration strategy, as the development of plant communities can improve soil physical structures, enhance accumulation of soil organic matter, and optimize the ecosystem services (Guo et al., 2018; Wang et al., 2019). Numerous studies have indeed been reported toward plant-mediated restoration of agricultural land in the United States (Alexander and Allan, 2007), Europe (Lamers et al., 2014), Brazil (Mendes et al., 2015), and China (Hou et al., 2019) through increased soil organic carbon (SOC) content and improved ecosystem function with increasing organic matter input and reducing disturbance (Li et al., 2007; Hou et al., 2010). In the context of background of sustainable development, the ecological restoration of degrade area has gradually raised people’s awareness and attracted extensive concerns across the world (Gong et al., 2013; Ilunga wa Ilunga et al., 2015).

The success of ecological restoration depends entirely on soil microbiomes that decompose plant-associated organic matters and improve soil physicochemical characteristics (Audino et al., 2014; Deng et al., 2020). Soil microorganisms are primary drivers of ecosystem processes in terms of nutrient cycling and energy transfer and significantly associated with regulating multiple ecosystem functions and enhancing ecosystem stability (Su et al., 2015). Soil microbial community structure and function are often considered to be sensitive to small variations in soil environment such as SOC and pH. Meanwhile, vegetation, land-use conversion, crop management, tillage intensity (Donnison et al., 2000; Pankhurst et al., 2002), and cultivation history (Bossio et al., 2005; Yao et al., 2006; Lauber et al., 2008) can also modify soil bacterial communities. Vegetation restoration with ryegrass (*Lolium perenne* L.) was more conducive to altering the microbial community structure than red clover (*Trifolium pratense* L.). However, understanding the diversity and function of soil microbial communities has been severely hampered because of the technical limitations in past decades because cultivation-dependent approaches can hardly represent what is naturally occurring under field conditions. The advent of high-throughput sequencing allows identification of keystone microbiomes in complex environments at unprecedented resolution in recent years. Guo et al. (2018) reported that natural revegetation of a semiarid habitat increased the microbial taxonomic diversity with plant diversity, and soil organic matter could explain most variations of microbial community structures. It has also been shown that the predominance of Acidobacteria was replaced by Proteobacteria after a 30-year land abandonment on the Loess Plateau (Zhang et al., 2016). However, contradictory observations have been reported, and it was often attributed to ecosystem-level heterogeneity. Meanwhile, the mere presence of functional genes does not necessitate metabolic activities of microbial communities in complex environments, although high-throughput sequencing can decipher taxonomic identities of total microbial communities at unprecedented level. Community-level physiological profiles (CLPPs) with Biolog EcoPlates can provide important insights into the functional diversity of soil microbial communities along distinct restoration scenarios (Rutgers et al., 2016). The microbial carbon metabolism represented by average well color development (AWCD) can be used to estimate the SOC mineralization. CLPPs are reproducible and sensitive to soil texture, soil type, tillage, land use, and vegetation restoration (Chou et al., 2016; Furtak et al., 2017). It has been shown that the increased bacterial diversity was likely associated with the reduced microbial carbohydrate catabolism under the biochar amendments (Chen et al., 2019). Grassland in the Netherlands and elsewhere in Europe tend to have higher functional diversity than do arable systems (Rutgers et al., 2016). Carbohydrates, amino acids, and carboxylic acids are more sensitive than other carbon sources as indicators of the health of black-soil grassland (Meng et al., 2008). However, to the best of our present understanding, there is no integrative study on both function (CLPP) and diversity (high-throughput sequencing) patterns of soil microbial communities in response to ecological restoration of degraded agricultural land.

Black soil, Udolls in the US Department of Agriculture (USDA) soil taxonomic system (Staff, 2014), is a typical soil characterized by high fertility and productivity, with native prairie in northeastern China representing 13.5% of the global area of black soil (IUSS Working Group WRB, 2007). In fact, this region is often considered as the most important agricultural land for crop production in China, particularly soybean and maize. The physicochemical properties of black soil, however, have changed drastically, especially SOC depletion, because agricultural land was reclaimed in the last hundred years. Numerous studies have shown significant deterioration of agricultural soils due to intensified anthropogenic interference, which poses great challenges for sustainable agriculture in China (Hao et al., 2017; You et al., 2019). Nonetheless, the microbial mechanisms underlying the restoration of agriculturally important lands are poorly understood. Therefore, four different treatments were established in 2003 on a degraded agricultural land for ecological restoration including (1) bare land (BL) control; (2) cropland (CL) control; (3) grassland (GL) for natural restoration; (4) alfalfa land (AL). A combination suite of techniques including classic functional approach (Biolog EcoPlates) and culture-independent molecular analysis [real-time quantitative polymerase chain reaction (PCR) and high-throughput sequencing] was then employed to decipher the keystone soil microbiome that are responsible for restoration of the degraded land under different planting regimes after a 14-year field management.

## Materials and Methods

### Site Description and Experiment Design

This work was carried out in the Hailun National Field Observation and Research Station (47°27’ N, 126°55’ E), Chinese Academy of Sciences, in Heilongjiang Province, China. This site has a typical continental monsoon climate. Mean annual rainfall is about 550 mm, with greater than 65% occurring between June and August. Mean annual temperature is 2.2°C, with mean monthly temperatures ranging from -23.5°C in January to 21.0°C in July. The area has a frost-free period of approximately 120 days. The soil is classified in the USDA soil taxonomy as an Udoll (Staff, 2014) derived from loamy loess, with a clay content of approximately 30% (Hao et al., 2017). The study site was a native prairie before crop cultivation about 200 years ago. No fertilizer was applied until cropping system was established 160 years ago. Chemical fertilizers (NPK) were used over 25 years before the setup of long-term experiments in 2003. Maize (*Zea mays* L.) as the major crop species is generally sown by row in May and harvested in October.

A long-term randomized-block field experiment was established in 2003, which consisted of three replicate plots of four treatments: (1) bare land (BL) control, the vegetation of wild grass was removed whenever emerged; (2) cropland (CL) control, maize (*Z. mays* L.) was planted without fertilizer application, and all aboveground biomass was removed; (3) grassland (GL) for natural restoration and *Poa annua* L., *Equisetum arvense* L., *Spodiopogon sibiricus* Trin. as the dominant species, the naturally occurring grass was left in the field; (4) alfalfa land (AL), the alfalfa (*Medicago sativa* L.) was planted, and the biomass was returned to the field. Each plot was 21.0 m^2^ (4.2 × 5 m) and isolated by cement barriers. Supplementary Figure S1 shows the change of the aboveground biomass of CL, BL, AL, and BL from 2004 to 2016 (Supplementary Figure S1).

### Sampling and Analysis

Five soil samples (0–20 cm) were randomly collected in each plot using a soil core auger (inner diameter of 7 cm) in July 2017 and then combined. After removing the visible plant debris manually, the samples were immediately transported to the laboratory for sieving through a 2-mm mesh and then stored at 4°C for studying the metabolism of C sources and at -80°C for subsequent molecular analysis. Subsamples were air-dried for analyzing pH and the SOC, total nitrogen (TN), exchangeable nitrogen (EN), and available phosphorus (AP) contents. pH was measured in a 2:1 water:soil suspension using a pH meter (Jackson, 1973). SOC and TN contents were determined using a VarioEL CHN elemental analyzer (Heraeus Ele-mentar VarioEL, Hanau, Germany). EN content was determined using alkaline diffusion (Page et al., 1982). AP content was determined as described by Olsen and Sommers (Olsen and Sommers, 1982).

### Community-Level Physiological Profiles

CLPPs were assessed using Biolog EcoPlates (Biolog Inc., CA, United States) containing 31 C substrates. Briefly, 5.0 g of soil was suspended in 45 mL of a sterile 0.85% NaCl solution, shaken for 30 min, and allowed to settle for 30 min. A 10-fold serial dilution up to 10^–3^ was then prepared. Each plate well was inoculated with 150 μL of the 10^–3^ dilution, and the plates were incubated in the dark at 25°C. The plates were read at 590 and 750 nm every 24 h for 192 h (8 days).

The optical densities (ODs) in each well were determined by subtracting the 750 nm values from the 590 nm values after correcting for the readings in a control well at the corresponding wavelengths. Negative ODs and ODs < 0.06 were set to zero (Classen et al., 2003). The use of six types of C sources was then statistically analyzed. The final values for each well after 8 days were used to calculate average well-color development (AWCD) (Garland and Mills, 1991):

$${\text{AWCD}}_{590-750\,n\,m}=\frac{\sum C_{590-750}}{31}$$

where 31 is the number of C sources used in the EcoPlates.

### Soil DNA Extraction

Microbial DNA was extracted from 0.5 g of fresh soil sample using an E.Z.N.A.^§^ soil DNA Extraction Kit (Omega Bio-tek, Norcross, United States) following the manufacturer’s instructions. The final DNA concentration and purity were determined using a NanoDrop 2000 UV-Vis spectrophotometer (Thermo Fisher Scientific, Wilmington, NC, United States), and the quality was checked by electrophoresis on an agarose gel (1%, wt/vol).

### Real-Time Quantitative PCR

The number of copies of the bacterial 16S rRNA gene in each sample was determined in triplicate using quantitative PCR (qPCR) in an ABI 7500 Real-Time PCR System (Applied Biosystems, Carlsbad, United States) with the 515F/907R primer set (5’-GTGCCAGCMGCCGCGGTAA-3’ and 5’-CCGTCAATTCMTTTRAGTTT-3’, respectively). Each PCR reaction contained 16.5 μL of 2 × SYBR Green qPCR Master Mix, 0.8 μL of 5 μM forward and reverse primers (each), 2.0 μL of template DNA, and ddH_2_O 33.0 μL. The PCR reactions were carried out at 95°C for 5 min, followed by 40 cycles of denaturation at 95°C for 30 s, annealing at 50°C for 30 s, and elongation at 72°C for 60 s. Negative controls contained all reagents, but the DNA was replaced with ddH_2_O. The threshold cycle (Ct) was obtained and averaged from triplicate samples. The number of copies of the 16S rRNA gene was calculated using a regression equation for converting Ct to the known number of copies in standard curves.

### Illumina MiSeq Sequencing

The 16S rRNA genes were amplified using the 515F and 907R primers in a PCR thermocycler (GeneAmp 9700, ABI). The reactions were performed in triplicate in 20-μL mixtures containing 4 μL of 5 × FastPfu Buffer, 2 μL of 2.5 mM dNTPs, 0.8 μL of each primer (5 μM), 0.4 μL of FastPfu Polymerase, 0.2 μL of bovine serum albumin, 10 ng of template DNA, and ddH_2_O for supplementing to the final volume. The reactions were conducted using the program: denaturation at 95°C for 3 min, 27 cycles of denaturation at 95°C for 30 s, annealing at 55°C for 30 s, and elongation at 72°C for 45 s, with a final extension at 72°C for 10 min. PCR products were extracted from a 2% (wt/vol) agarose gel after electrophoresis, purified using an AxyPrep DNA Gel Extraction Kit (Axygen Biosciences, Union City, United States), and quantified using QuantiFluor^§^ -ST (Promega, United States) following the manufacturer’s instructions. Purified amplicons were pooled in equimolar concentrations and paired-end sequenced (2 × 300) on an Illumina MiSeq platform (Illumina, San Diego, CA, United States) following standard protocols provided by Majorbio Bio-Pharm Technology Co. Ltd. (Shanghai, China). The raw reads were deposited in the NCBI Sequence Read Archive database (accession no. PRJNA588746).

Raw sequence data were demultiplexed, filtered for quality using Trimmomatic, and merged using FLASH with the criteria: (1) the reads were truncated at sites with average quality scores < 20 over a 50 bp sliding window; (2) the primers were matched exactly, allowing mismatches of two nucleotides, and reads containing ambiguous bases were removed; and (3) sequences with overlaps > 10 bp were merged based on their overlap sequence. Operational taxonomic units (OTUs) were clustered with a similarity cutoff of 97% using UPARSE (version7.1^1^; Edgar, 2013), and chimeric sequences were identified and removed using UCHIME (Edgar et al., 2011). The clean reads were assigned to the OTUs using the Silva (SSU123) 16S rRNA database and the RDP classifier algorithm (Quast et al., 2013). The data were analyzed using the free online Majorbio I-Sanger Cloud Platform^2^. Keystone taxa are defined as the phylotypes whose relative abundance showed statistically significant increase in soils of CL (A), GL (B), and AL (C) when compared with control bare soil BL. Meanwhile, the phylotype showing significant decline in GL and AL soils could be considered as indicative taxa of soil degradation.

### Statistical Analysis

Multiple comparisons of Duncan multiple range tests were used to identify significant changes in the number of copies of the 16S rRNA gene and significant changes to groups at the phylum level (*P* < 0.05) (SPSS Inc., United States). A principal component analysis (PCA) conducted using the vegan (2.5–6) package was used to analyze the characteristics of C-source metabolism at the community level. CL, GL, and AL were compared to BL at the genus level using STAMP (v2.1.4) *t*-tests to identify keystone taxa (genera) with significant changes in relative abundances (*P* < 0.05) (Parks et al., 2014). A principal coordinate analysis (PCoA) based on Jaccard and Bray–Curtis distances was performed at the OTU level for all samples. In order to examine the effects of ecological restoration on microbial community, analysis of similarities (ANOSIM) (Clarke and Ainsworth, 1993) was performed. Correlations among the treatments were estimated using the Bray–Curtis distances to cluster all samples. A redundancy analysis (RDA) was performed to estimate the correlations between the microbial communities and soil properties. Pearson correlation was used to determine the relationships between soil properties and microbial community characteristics. PCA, ANOSIM, and RDA were carried out within the R (3.4.1). The functions from the prokaryotic clades were predicted using FAPROTAX (Louca et al., 2016).

## Results

### Plant Aboveground Biomass and Soil Properties

The BL soil received no plant biomass in the past 14 years (Table 1). The CL plots with maize generated the highest aboveground biomass about 24.80 kg plot^–1^ every year (Table 1), but all of the aboveground biomass was removed from the field (Supplementary Figure S1). The annual incorporation of average aboveground biomass from 2004 to 2016 into soils of GL and AL was 10.00 and 9.67 kg plot^–1^, respectively. The four long-term treatments have resulted in significant changes of soil properties (Table 1), which was closely related with the input of plant biomass (Supplementary Figure S1).

**TABLE 1:** Soil properties and plant characteristics under different restoration managements of vegetation scenarios.

	**Items**	**BL**	**CL**	**GL**	**AL**
Soil	SOC (g ⋅ kg^–1^)	26.29 ± 0.30^*c*^	26.77 ± 0.58^*c*^	35.85 ± 1.22^*a*^	32.77 ± 0.28^*b*^
	pH	5.75 ± 0.05^*d*^	6.15 ± 0.01^*b*^	6.33 ± 0.04^*a*^	5.91 ± 0.07^*c*^
	TN (g ⋅ kg^–1^)	1.87 ± 0.03^*b*^	1.98 ± 0.10^*b*^	2.62 ± 0.12^*a*^	2.51 ± 0.07^*a*^
	EN (mg ⋅ kg^–1^)	133.04 ± 3.72^*c*^	137.21 ± 6.76^*c*^	169.93 ± 3.57^*a*^	159.82 ± 4.49^*b*^
	AP (mg ⋅ kg^–1^)	26.55 ± 0.56^*c*^	16.01 ± 3.56^*d*^	49.78 ± 2.48^*a*^	43.11 ± 3.45^*b*^
	C/N	14.04 ± 0.13^*a*^	13.53 ± 0.93^*a*^	13.67 ± 0.26^*a*^	13.04 ± 0.25^*a*^
Plant	Biomass (kg ⋅ plot^–1^)	—	24.80 ± 1.12^*a*^	10.00 ± 1.76^*b*^	9.67 ± 0.67^*b*^
	C (g ⋅ kg^–1^)	—	45.49 ± 0.42^*b*^	44.30 ± 0.35^*c*^	47.03 ± 0.60^*a*^
	N (g ⋅ kg^–1^)	—	0.93 ± 0.06^*c*^	1.55 ± 0.06^*b*^	2.02 ± 0.06^*a*^
	C:N	—	49.28 ± 3.62^*a*^	28.66 ± 0.85^*b*^	23.33 ± 0.81^*c*^

**BL, bare land; CL, cropland; GL, grassland; AL, alfalfa land. SOC, soil organic carbon; TN, total nitrogen; EN, exchangeable nitrogen; AP, available phosphorus. Values are means ± standard error (n = 3). Different letters (a–d) represent significant differences by Duncan multiple-range test at P < 0.05. Biomass was the average value of the samples from 2003 to 2016, and the aboveground C and N were from the samples of 2016. The underlined value means the aboveground biomass was removed, C, N, and C:N. — stands for not determined.**

Compared with BL and CL, the content of SOC in GL and AL was significantly increased by 6.00–9.56 g/kg (*P* < 0.05). Similarly, the TN, EN, and AP contents of GL and AL were higher than those in BL and CL soils. The SOC, TN, and EN contents in CL treatment were not significantly higher than those in BL soil. Soil AP content was lower in CL relative to BL, and the C:N ratio showed no significant differences among all the treatments. Soil pH values were the highest in GL soil (6.33) and the lowest in BL soil (5.75).

### Community Metabolisms of Carbon Substrates in Soil

**FIGURE 1:**
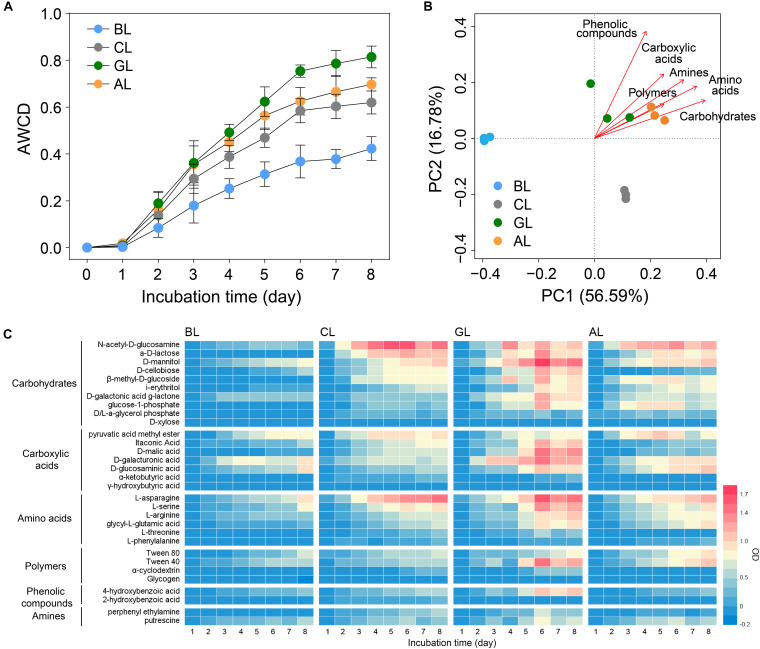
Community-level patterns of microbial carbon metabolism by Biolog EcoPlate in soils under different restoration managements. **(A)** Carbon utilization intensities for six different substrates of carbon compounds as indicated by AWCD values (average well color development) in soils; **(B)** principal component analysis (PCA) showing the community-level physiological profiling patterns of carbon utilization in different soils; **(C)** temporal dynamics of carbon utilization intensity on 31 different substrates (Biolog EcoPlate) by microbial communities in soils under different restoration managements. BL, bare land; CL, cropland; GL, grassland; AL, alfalfa plantation.

The GL and AL had the higher AWCDs throughout the incubation period (*P* < 0.05) (Figure 1A). PC1 and PC2 explained 56.59 and 16.78% of the variance in the data, respectively (Figure 1B). The treatments were divided into three groups based on the PCA scores. GL and AL treatments were grouped in the first quadrant; CL and BL treatments were separated in the other quadrants (Figure 1B). The results from the sixth day of incubation showed that the relative utilization rates of carbohydrates (*r*^2^ = 0.6205, *P* < 0.01), phenolic compounds (*r*^2^ = 0.6411, *P* < 0.01), amino acids (*r*^2^ = 0.5947, *P* < 0.017), and amines (*r*^2^ = 0.5175, *P* < 0.05) were stimulated significantly in GL and AL treatments relative to CL and BL soils (Figure 1C). Although the GL and AL treatments had similar metabolic patterns, the colors on substrates D-mannitol, D-cellobiose, D-galactonic acid g-lactone, itaconic acid, D-malic acid, D-galacturonic acid, L-serine, Tween 40, and 4-hydroxybenzoic acid were more intensive in GL than that in AL (*P* < 0.05) (Figures 1B,C).

### Population Dynamic of Microbial Communities in Soil

**FIGURE 2:**
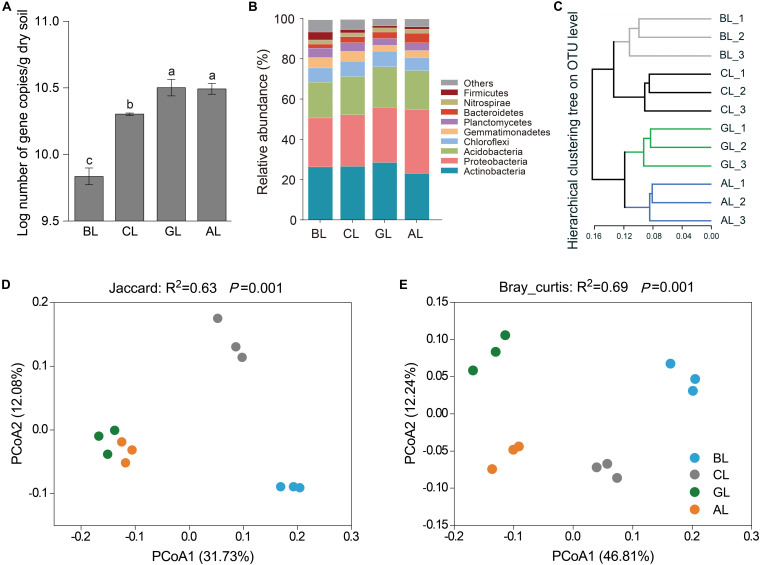
Community divergence of soil microbiomes under different restoration managements. **(A)** The abundance of bacterial 16S rRNA gene copies in soils; **(B)** relative abundances of different bacterial phyla in soils; **(C)** hierarchical clustering of microbial community structures based on Bray–Curtis matrix at OTUs level. Beta diversity by Jaccard **(D)** Bray–Curtis **(E)** matrix showing community shift in different soils at OTUs level.

**FIGURE 3:**
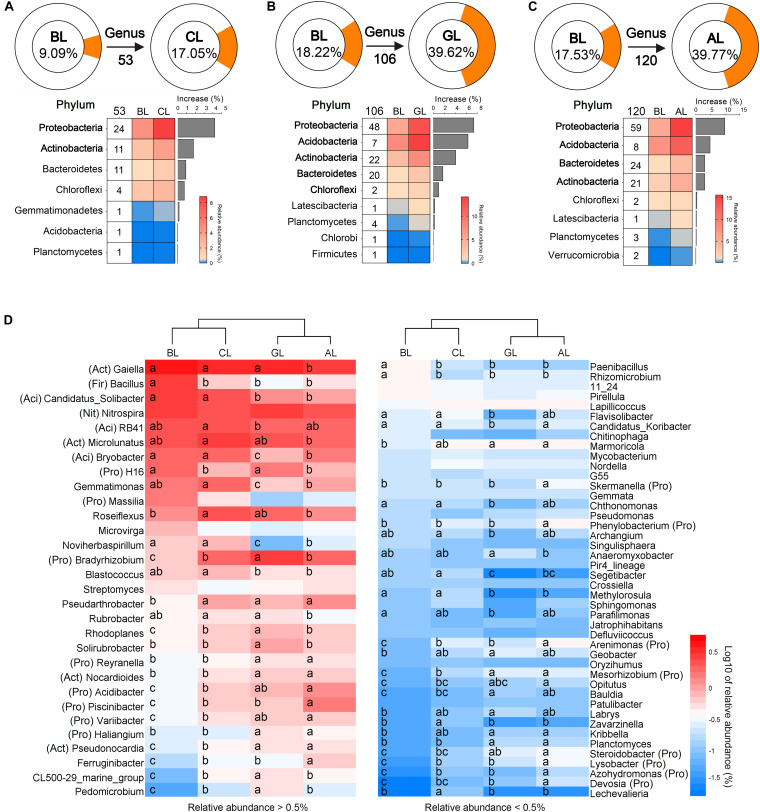
Keystone taxa showing significant changes at genus level in soils under different restoration managements. Keystone taxa are defined as the phylotype whose relative abundance showed statistically significant increase in soils of CL **(A)**, GL **(B)**, and AL **(C)** when compared with control bare soil BL. The portion in brown of the ring diagram represents total abundance of all keystone taxa, and the value below the arrow indicates the number of keystone taxa. These keystone genera are further classified into different phyla in the heatmap under the ring diagram. For example, a total of 53 genera of 7 phyla in CL soil showed significant increase relative to BL soil, and the total abundance of these 53 genera is 9.09 and 17.05% in BL and CL soils, respectively. The net increase in relative abundance of all 24 keystone genera within Proteobacteria phylum in CL soil is 4.23% when compared to those in BL soil. **(D)** Changes in relative abundances of keystone taxa in soils under different restoration managements. The heatmap in left and right sides represents the keystone genera with relative abundance greater and less than 0.5%, respectively. Different letters (a–d) represent significant differences by Duncan multiple-range test at *P* < 0.05.

High-throughput sequencing resulted in high-quality sequences from 19,159 to 25,431 for each sample, and 19, 159 sequences were selected for normalization in each sample for subsequent analysis. The keystone taxa were defined as the phylum or genus showing statistically significant increase in relative abundance in soil samples of CL and GL relative to BL control soils (Figure 3). Real-time qPCR revealed higher copy numbers of the 16S rRNA genes in GL and AL than that in BL treatment (Figure 2A).

Bacteroidetes was the only keystone phylum with significant higher abundance in AL (4.81%) than other treatments (*P* < 0.05) as shown in Figure 2B. At the level of phylum, the dominant bacterial groups were Proteobacteria, Actinobacteria, Acidobacteria, and Chloroflexi with a relative abundance of > 5% in all three land use types (Figure 2B). The most dominant bacterial phyla were Proteobacteria (31.73%) in AL treatment and Actinobacteria (26.38–28.53%) in the other treatments (Figure 2B). No statistically significant difference in abundance of Acidobacteria was discovered among all the treatments (*P* > 0.01). The relative abundances of Firmicutes and Cyanobacteria were significantly declined by 74.53 and 84.71% in GL and by 68.63 and 69.42% in AL, respectively (*P* < 0.05), compared to that in BL (Supplementary Figure S2). The relative abundances of Gemmatimonadetes were not significantly different between CL and BL, and they were significantly reduced in AL and GL, respectively, compared to that in BL. Hierarchical clustering and a PCoA plot with Bray_curtis matrix demonstrated that the microbial communities from the four treatments formed distinct groups (Figure 2C), which was further supported by beta diversity by Jaccard (Figure 2D) Bray–Curtis (Figure 2E) matrix showing community shift in different soils at OTU level.

We identified indicative genera during ecological restoration by changes in relative abundance. A total of 490 genera were detected in samples among all treatments (Figure 3), and the relative abundances of 53, 106, and 120 genera were significantly increased in soils of CL (Figure 3A), GL (Figure 3B), and AL (Figure 3C) treatments, respectively, compared to that in BL soil. Meanwhile, 32, 57, and 39 genera showed significant declines in the soils of CL, GL, and AL treatments, respectively. In GL and AL, the number of group with significantly increased relative abundance at the phylum level was higher than CL. Proteobacteria was the phylum with the highest relative abundance increase in all treatments. The genera with the highest increase in relative abundance were Bradyrhizobium, Ramlibacter, Piscinibacter, and Roseiflexus in CL and Bradyrhizobium, Solirubrobacter, Nocardioides, Acidibacter, and Rhodoplanes in GL and Bradyrhizobium, Acidibacter, Pseudarthrobacter, Nocardioides, and Flavobacterium AL. Clustering analysis further revealed that the keystone genera classified GL and AL soil into one group, whereas CL and BL formed the other group, regardless of the relative abundance of greater or less than 0.5%, respectively (Figure 3D). Among these enriched genera, *Gaiella*, *Microlunatus*, *RB41*, *Candidatus Solibacter*, *Bryobacter*, *Nitrospira*, and *Roseiflexus* were dominant in all treatments (Figure 3D). The lowest relative abundance of *Bradyrhizobium* (0.76%) and the highest relative abundance of *Bacillus* (2.35%), *Candidatu Solibacter* (2.11%), *Massilia* (1.16%), *Rhizomicrobium*, and *H16* were observed in the BL treatment. *Reyranella*, *Mesorhizobium*, *Devosia*, *Haliangium*, *Nocardioides*, and *Pseudonocardia* were significantly more abundant in GL and AL than that in CL and BL. *Variibacter*, *Phenylobacterium*, *Skermanella*, *Piscinibacter*, *Azohydromonas*, *Acidibacter*, *Arenimonas*, *Steroidobacter*, and *Lysobacter* were significantly more abundant only in AL, while less abundant in BL. *Bacillus* were reduced 2. 05-, 5. 15-, and 3.57-fold in the CL, GL, and AL, respectively, compared to that in BL.

### Relationships Between Microbial Community Composition and Soil Properties

**FIGURE 4:**
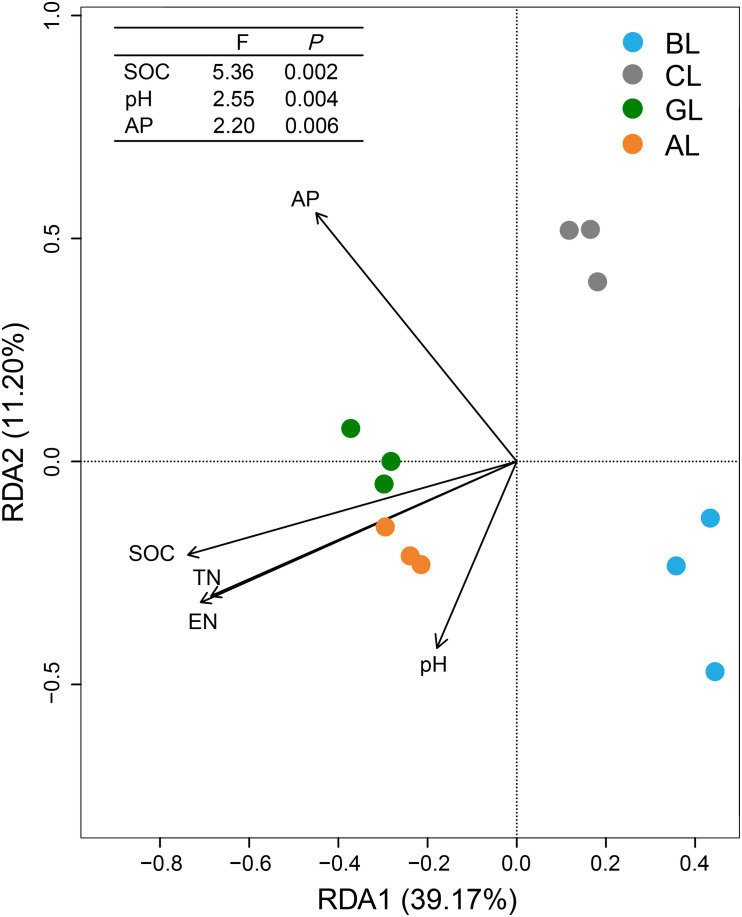
Redundancy analysis (RDA) showing relationships between soil physiochemical characteristics and microbial communities. SOC, soil organic carbon; TN, total nitrogen; EN, exchangeable nitrogen; AP, available phosphorus.

The overall structures of microbial communities in different treatments were significantly linked to the soil properties (Figure 4). RDA ordination analysis resulted in three distinct groups. The treatments of AL and GL were grouped into a cluster, whereas the treatments of BL and CL formed two control clusters, respectively. The main factors that grouped the treatments of AL and GL were SOC, TN, and EN. Meanwhile, cluster of BL samples appeared to be mostly influenced by pH. Cluster of CL samples in turn was significantly affected by the content of AP. SOC content was the most influential soil property (*P* < 0.01) for the microbial communities, followed by pH (*P* < 0.01) and AP content (*P* < 0.01).

## Discussion

The purpose of the present study was to decipher the keystone taxa showing significant increases in the degraded agricultural soil under different restoration regimes and to elucidate the changes in soil characteristics that could have affected community-level carbon metabolisms and keystone microbiomes in soils during a 14-year field experiment. Our results provided compelling evidences that the functional activity and composition of soil microbial communities during the restoration of BL to CL, AL, and GL were driven by changes in soil properties that were directly associated with the input and decomposition of plant biomass.

### Restoration of Soil Fertility Under Different Vegetation Regimes

Our study revealed that the selected soil properties SOC, pH, TN, EN, and AP were improved during the conversion from BL to CL, AL, and GL (*P* < 0.05). Our study showed that GL and AL had greater SOC, pH, TN, EN, and AP content than those in BL and CL. The SOC and TN content in AL increased by 24.65 and 36.36% and in GL by 34.22 and 40.10%, respectively, when compared to that in BL. In our study, a large amount of aboveground biomass of grass (10.0 kg plot^–1^) and alfalfa (9.67 kg plot^–1^) were input to the GL and AL, respectively, whereas those were moved out from the BL and CL (Table 1 and Supplementary Figure S1). In addition, annual crops invest less in root systems than do the perennial plants, with root biomass generally accounting for 5–30% of the total plant biomass for many crops, including wheat and maize (Wu et al., 2011; Wang et al., 2014). Thus, the improved content of SOC might be attributed to the recovery of aboveground and underground biomass of plants and the decrease of tillage disturbance, which directly increase the inputs of organic C from aboveground tissues, roots, and root exudates into the soil and reduce the loss of SOC caused by disturbance of the soil (Romkens et al., 1999; Hao et al., 2017; Kantola et al., 2017; Liao et al., 2018). This result is largely consistent with that of Machmuller et al. (2015) in the southeastern United States, who found similar increases in SOC content after 6 years of changing a cropland to grassland. The grassland GL soil had significantly higher SOC contents than that of alfalfa after 14 years of experiment in the present study. By contrast, no significant difference in SOC contents was found in the conversion from cropland to alfalfa and grass for 2 years (Yu et al., 2017). Thus, the restoration duration is also an important factor affecting the soil nutrient contents (Guo et al., 2018). The lowest pH was observed in BL, which could be explained by the excessive application of mineral fertilizers and the continuous removal of base cations by crop harvest (Guo et al., 2010). Compared to BL, soil pH in our study was also significantly higher in GL and AL, perhaps due to the accumulation of large quantities of biomass as well (Bolan et al., 1991). Differences among the soil properties induced by distinct cover crop influence the structures of microbial communities (Lauber et al., 2008). Therefore, vegetation has played an important role in controlling cropland degradation through adjusting soil nutrient level (such as SOC and pH) in favor of microbial growth (Singh and Singh, 2006; Shrestha and Lal, 2010).

### Identification of Keystone Taxa in Soils Under Different Restoration Regimes

The changed soil microbial community structure in AL and GL were attributed to changes in soil properties and vegetation, which could alter the microenvironment of microbes in soil (Finney et al., 2017). It has been reported that high plant diversity could increase the entering of different litters and root exudates to the soil and generate higher amounts of readily accessible C substrate to feed soil microorganisms (Wardle, 2006; Zhu et al., 2010).

Bacteroidetes is the only keystone taxa at phylum level showing significant increase in relative abundance in AL soils when compared to other treatments (Figure 2B). It includes 24 keystone genera (Figure 3C). For example, relative abundance of *Ferruginibacter* was 0.186% in BL, whereas relative abundance of these genera increased by more than two times in CL and GL and more than three times in AL. Relative abundance of *Segetibacter* was 0.15 and 0.19% in BL and CL. However, relative abundance of *Segetibacter* was only 0.02 and 0.05% in GL and AL. This result may reflect the fact that each microbial group has the best adapted environment and with capacity to bloom under favorable conditions. At the level of phylum, ecological restoration did not change the dominant phyla of the soil bacterial community, but their relative abundances were significantly altered (Figure 2B and Supplementary Figure S2). The dominant bacterial phyla, including Proteobacteria, Actinobacteria, and Acidobacteria, were in line with those in previous studies (Liu et al., 2014; Ding et al., 2016; Chen et al., 2020). Compared with the BL community, the relative abundance of Proteobacteria increased by 30.42%, whereas that of Actinobacteria decreased by 12.36% in the AL, and the relative abundance of Proteobacteria and Actinobacteria increased by 13.19 and 8.15% in the GL, respectively. Proteobacteria and Actinobacteria are usually regarded as copiotrophic microbes that existed in soil under sufficient nutrient conditions, and they play important roles in the soil carbon cycle (Fierer et al., 2007; Zhang et al., 2016). Thus, it can be concluded that the transition in the relative abundance of Proteobacteria and Actinobacteria after land-use conversion could be a result of increase in soil nutrients (SOC and pH) (Table 1, Figure 4, and Supplementary Table S2). In the present study, restoration (GL and AL) decreased the relative abundances of Cyanobacteria and Firmicutes compared with that in BL. Bacteria in these two phyla are commonly believed as oligotrophic groups occurring in low nutrients and disturbed soil (De Deyn and Van Der Putten, 2005; Barnard et al., 2013). In contrast, Trivedi et al. (2016) summarized previous studies and indicated that natural revegetation increases abundance of Cyanobacteria and decreases abundance of Firmicutes. The decrease of Cyanobacteria (photoautotrophs) could be related to the decrease of sunlight availability in soil due the increasing vegetation coverage (Zhang et al., 2018). Additionally, previous report showed that alfalfa cultivation can decrease the number and Shannon index of Cyanobacteria species in soil, because alfalfa roots release various flavonoids that can substantially affect growth and physiological functions of Cyanobacteria species (Huang et al., 2015; Alghanmi and Jawad, 2019). Lower photosynthetic carbon sequestration group (belonging to Cyanobacteria) appeared in GL and AL 4.78- and 2.25-fold, respectively, greater than that in BL (Supplementary Table S1). Therefore, further researches are needed to address the discrepancy, which might probably be attributed to the different vegetation restoration types, climate, and soil fertility.

A number of genera within dominant phyla were identified as keystone taxa, although no significant increase was observed for the dominant taxa at phylum level except for Bacteroidetes. A number of N_2_ fixers were found to be keystone genera showing significantly higher abundance in GL and AL soils than BL and CL soils, including *Bradyrhizobium*, *Devosia*, *Mesorhizobium*, and *Azohydromonas.* These *nif* gene–containing keystone taxa can fix nitrogen and promote plants growth (Franche et al., 2009; Martínez-Hidalgo and Hirsch, 2017). In addition, the quality and quantity of aboveground litter and belowground roots supplied to the soil differed greatly in GL and AL and presumably stimulated the abundance of *Nocardioides* significantly in association with the accelerated decomposition of xylan (Whitman et al., 2012). *Steroidobacter* was significantly more abundant in AL treatment and may contribute to the mineralization of soil organic matter to meet the N demand of plants (Lian et al., 2017). Alfalfa, a leguminous plant, can affect the nitrogen cycle in soil. The genera associated with the nitrogen cycle (Garrity, 2005; Fahrbach et al., 2008; Yuan et al., 2014), such as *Skermanella*, were highly abundant only in AL treatment (Figure 3D). The relative abundances of *Bacillus*, *Rhizomicrobium*, and *Paenibacillus* were significantly higher in BL treatment than in other treatments. All of these genera were capable of diverse functions, such as Plant Growth Promoting Rhizobacteria (PGPR), nitrogen fixation, and litter decomposition. In fact, *Bacillus* members are well known to survive in harsh environments (Tao et al., 2015). We speculated that the oligotrophic environment in BL could have selectively stimulated the growth and enhanced the relative abundance of *Bacillus*. Compared with that in BL soil, the relative abundance of *Bacillus* was lower in CL, GL, and AL treatments 2. 05-, 5. 15-, and 3.57-fold, respectively (Figure 3D). It thus implies that *Bacillus* might serve as an indicator of agricultural land degradation. Stable-isotope probing of functional processes such as nitrogen fixation would be of help toward better understandings of dynamic changes of keystone taxa in soils such as *Rhizomicrobium* and *Paenibacillus* under distinct restoration scenario of the degraded agricultural land.

### Community Metabolism of Carbon Substrates in Soils

The land restoration not only selected for distinct keystone microbiomes but also affected metabolic functions of microbial communities in soils. The C metabolism (Figure 1) of total bacteria in GL and AL treatments was significantly higher than those in CL and BL treatments. Soil microbial communities in GL and AL exhibited CLPPs different from those in CL and BL, indicating their different C substrate preferences (Figure 1B). The microbial communities in GL and AL were more efficient in using carbohydrates, phenolic compounds, amino acids, and amines than those in BL and CL. The potential causes of this discrepancy are the following: (1) the different inputs of aboveground/root biomass and type/amount of root exudates could affect the total abundance of microbial communities (Figure 2A), which in turn lead to distinct decomposition capability in various treatments; (2) disturbed agricultural managements or tillage could affect the heterotrophic activities of soil microbial communities by altering the aeration of the soil body and contact between the SOC and microbes. Under the condition of undisturbed ecological recovery, the plant microbial community interaction was stronger in GL and AL than that in CL and BL soils, because the plant residues in the soil stimulated the proliferation of chemoheterotrophic groups, which increased the metabolic capacity of the microbial community and ultimately improved the soil health and ecosystem function (Alcoze et al., 2000; Izquierdo et al., 2005). In GL treatment, the utilization intensity of D-cellobiose, itaconic acid, D-malic acid, and 4-hydroxybenzoic acid was higher in GL than AL, which can be attributed to the higher plant diversity of grassland. In addition, the relative abundance of chemoheterotrophic microbes in GL was significantly higher than that in AL (Supplementary Table S1). The nutrient contents of BL as a degraded soil were not different from that of CL treatment, whereas the metabolic capacity, the amount of carbon source utilization, and the relative abundance of chemoheterotrophic microbes in CL treatment were significantly higher than those in BL treatment, indicating that CLPP can be a useful indicator of soil degradation/restoration. Further studies linking the specific physiology of carbon metabolism to the taxonomic identity of active keystone taxa are warranted.

### Linking Soil Characteristics With Keystone Taxa in Soils

In the present study, keystone taxa were identified (Figure 3), and dynamic changes of keystone microbiomes were assessed in association with multiple environmental factors (Figure 4 and Supplementary Table S2). RDA ordination indicated that soil SOC content, pH, and AP played dominant roles in shaping the microbial community composition (Figure 4). This observation was contradictory to previous findings that pH was the key factor regulating the structures of bacterial community in soils (Cao et al., 2017), both on large and fine scales (Shen et al., 2013). Meanwhile, it has been shown that SOC was the best predictor for microbial community structure in soils across different agricultural land management at a given location (Sul et al., 2013). Our study revealed that the relative abundances of Proteobacteria, Firmicutes, and Cyanobacteria were significantly correlated with most of the measured soil properties (Supplementary Table S2). The soil characteristics SOC, TN, and EN had significantly positive correlations with Proteobacteria and had no significant correlations with Actinobacteria and Acidobacteria (Supplementary Table S2). Cyanobacteria and Firmicutes are the less abundant bacterial phyla in terrestrial habitats and are negatively related with SOC, pH, TN, and EN (Supplementary Table S2). Firmicutes are very important to decompose the vegetable litter (Nuccio et al., 2013; Cui et al., 2019). Compared to BL soil, GL and AL soils showed dramatic decrease in abundance of Firmicutes, implying that the members of which were not important in the composition of plant residues/litters, being consistent with the recent findings (Cui et al., 2019) that microbial community shifts during the conversion of the cropland to brushland. Despite the correlative analysis, long-term field experiment represents a net result of microbial community changes over 14 years and very likely reflects what is occurring under the field conditions. But it remains unclear to what extent these decreasing phylotypes could represent the progressive degradation of agricultural land under intensive anthropogenic interference. Future studies with isolates of target keystone taxa can provide more information on the ecophysiology of functional microbiome in restoring the degraded agricultural land.

## Conclusion

This study demonstrated that grassland vegetation (GL) and alfalfa planting (AL) could significantly enhance soil fertility after 14 years of ecological restoration. The relative abundances of dominant phyla showed significant changes, although the composition of soil microbiome remained largely unchanged. The relative abundance of the key taxa, such as genera of *Reyranella*, *Mesorhizobium*, *Devosia*, *Haliangium*, *Nocardioides*, and *Pseudonocardia*, were significantly more abundant in GL and AL. The relative abundance of *Bacillus* was significantly higher in the BL than other treatments. *Bacillus* (Firmicutes) and Cyanobacteria genus showed significant decrease in abundance in the GL and AL soils when compared to BL soil. These two taxa can be considered as bioindicators of agricultural land degradation. Furthermore, RDA and correlation analysis revealed that the SOC content was the primary factor determining the population dynamics changes of keystone microbiomes during ecological restoration. Overall, this study provides insights into the keystone microbiomes associated with decomposition of plant biomass and the carbon source metabolism of microbial communities in degraded black soil under different restoration scenarios in northeastern China.

## Data Availability Statement

The datasets generated for this study can be found in the NCBI under accession number: PRJNA588746.

## Author Contributions

JY designed the study. ZZ and XC performed the work. XH and XL analyzed the data. EW and WZ revised the manuscript. All authors read and approved the final manuscript.

## Conflict of Interest

The authors declare that the research was conducted in the absence of any commercial or financial relationships that could be construed as a potential conflict of interest.

## Footnotes

1 http://drive5.com/uparse/

2 http://www.i-sanger.com

## Supplementary Material

FIGURE S1 The annual input of aboveground biomass under different restoration of vegetation managements from 2004 to 2016. The abbreviation of BL denotes that the degraded bare land receiving no plant-associated matter as the vegetation of wild grass was removed whenever emerged. The abbreviation of CL indicates that the maize (*Zea mays* L.) land receiving no fertilizer andall aboveground biomass of maize was removed every year. The abbreviations of GL and AL represent the vegetation treatments of natural grassland and alfalfa plantation, and the biomass of 10.00 and 9.67 kg plot^–1^, respectively, were returned to the field for natural restoration and alfalfa plantation. The CL_(1) refers to the healthy land with maize crop under traditional fertilization managements, and it shows the highest aboveground biomass about 42.74 kg plot^–1^ every year. BL, bare land; CL, cropland; CL_(1), cropland with traditional fertilization management; GL, grassland; AL, alfalfa plantation.

FIGURE S2 Relative abundances of bacterial phyla under different restoration managements. Different letters (a-c) represent significant differences by Duncan’s multiple range test at *P* < 0.05.

TABLE S1 Relative abundance of predicted function groups based on FAPROTAX database.

TABLE S2 Correlations between soil properties and the relative abundance of the main phyla and genera.

## Abbreviations

AL: Alfalfa land
AP: Available phosphorus
AWCD: Average well-color development
BL: Bare land
CL: Cropland
CLPP: Community level physiological profiles
EN: Exchangeable nitrogen
GL: Grassland
OD: Optical density
OTUs: Operational taxonomic units
PCA: Principal Component Analysis
PCoA: Principal coordinate analysis
qPCR: Quantitative real-time PCR
RDA: Redundancy analysis
SOC: Soil organic carbon
TN: Total nitrogen.
