# Supplementary material for: Keystone Microbiomes Revealed by 14 Years of Field Restoration of the Degraded Agricultural Soil Under Distinct Vegetation Scenarios
Source: Front Microbiol. 2020 Aug 18;11:1915. doi: 10.3389/fmicb.2020.01915 (PMC7461875; doi:10.3389/fmicb.2020.01915)
Supplement: FIGURE S1 — The annual input of aboveground biomass under different restoration of vegetation managements from 2004 to 2016. The abbreviation of BL denotes that the degraded bare land receiving no plant-associated matter as the vegetation of wild grass was removed whenever emerged. The abbreviation of CL indicates that the maize (Zea mays L.) land receiving no fertilizer andall aboveground biomass of maize was removed every year. The abbreviations of GL and AL represent the vegetation treatments of natural grassland and alfalfa plantation, and the biomass of 10.00 and 9.67 kg plot–1, respectively, were returned to the field for natural restoration and alfalfa plantation. The CL_(1) refers to the healthy land with maize crop under traditional fertilization managements, and it shows the highest aboveground biomass about 42.74 kg plot–1 every year. BL, bare land; CL, cropland; CL_(1), cropland with traditional fertilization management; GL, grassland; AL, alfalfa plantation. [file Data_Sheet_1.docx]

Supplementary Material


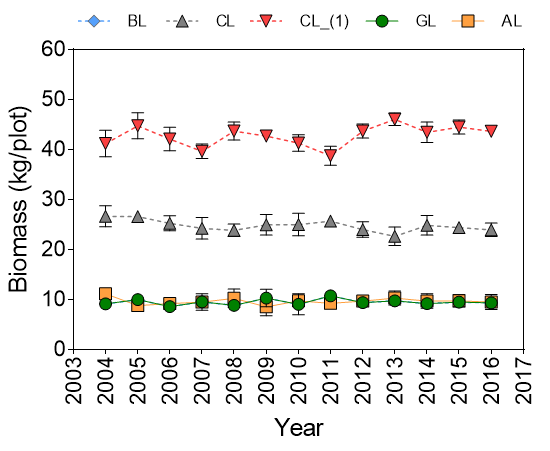


**Supplementary FIGURE 1** **ǀ** The annual input of aboveground biomass under different restoration of vegetation managements from 2004 to 2016. The abbreviation of BL denotes that the degraded bare land receiving no plant-associated matter as the vegetation of wild grass was removed whenever emerged. The abbreviation of CL indicates that the maize (*Zea mays* L.) land receiving no fertilizer andall aboveground biomass of maize was removed every year. The abbreviations of GL and AL represent the vegetation treatments of natural grassland and alfalfa plantation, and the biomass of 10.00 and 9.67 kg plot^-1^, respectively, were returned to the field for natural restoration and alfalfa plantation. The CL_(1) refers to the healthy land with maize crop under traditional fertilization managements, and it shows the highest aboveground biomass about 42.74 kg plot^-1^ every year. BL, bare land; CL, cropland; CL_(1), cropland with traditional fertilization management; GL, grassland; AL, alfalfa plantation.


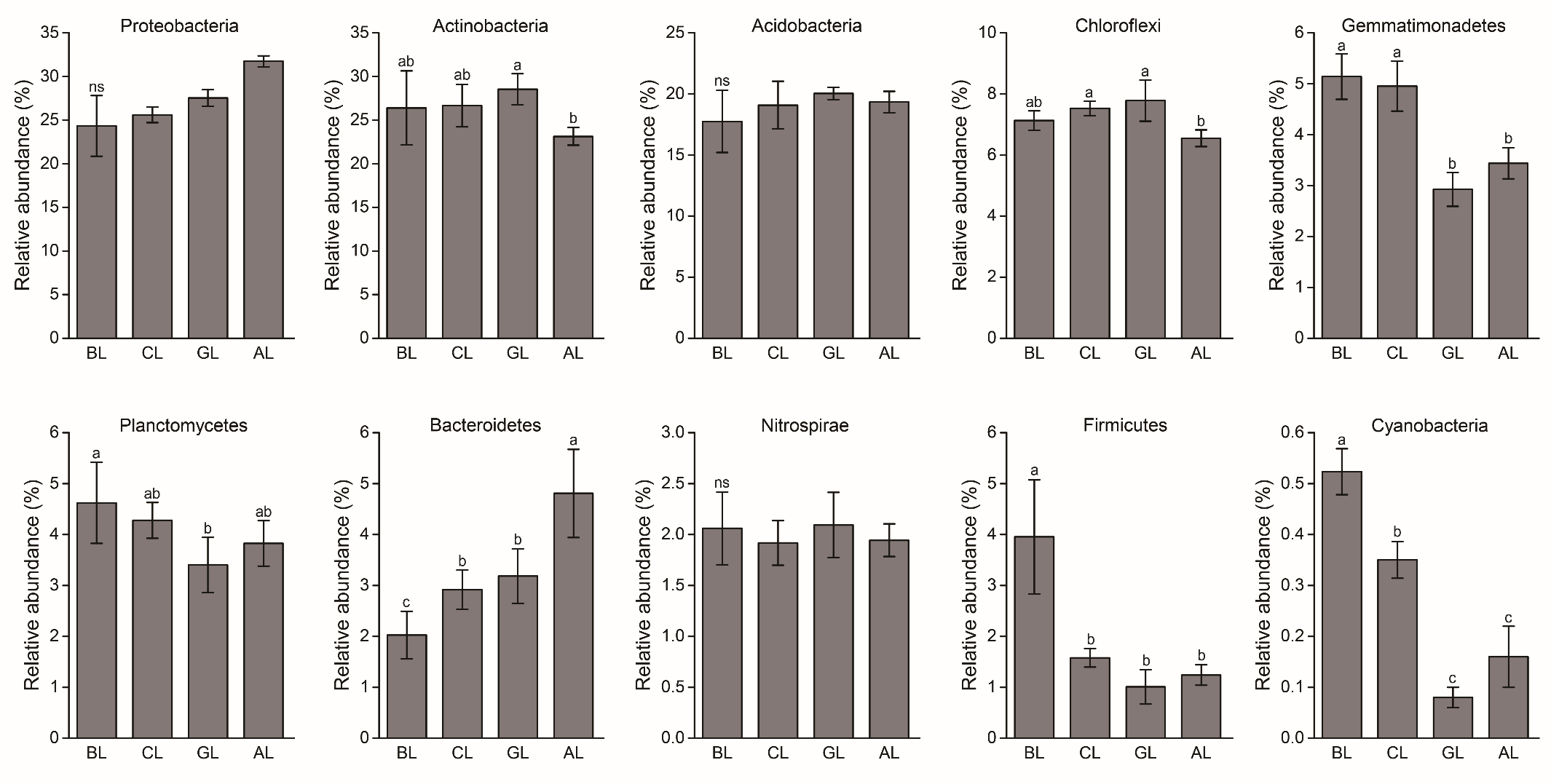


**Supplementary FIGURE 2 ǀ** Relative abundances of bacterial phyla under different restoration managements. Different letters (a-c) represent significant differences by Duncan’s multiple range test at *P* < 0.05.

**Supplementary TABLE 1 ǀ** Relative abundance of predicted function groups based on FAPROTAX database.

| Function groups | Phylum | BL | CL | GL | AL |
| --- | --- | --- | --- | --- | --- |
| Phototrophy | *Cyanobacteria* | 0.52 a | 0.32 b | 0.09 c | 0.16 c |
| Chemoheterotrophy | *Euryarchaeota* | 0.05 | 0.02 | 0.00 | 0.00 |
|  | *Acidobacteria* | 1.42 | 1.39 | 0.90 | 1.14 |
|  | *Proteobacteria* | 0.68 | 1.25 | 2.13 | 2.03 |
|  | *Bacteroidetes* | 0.19 | 0.39 | 0.39 | 0.67 |
|  | *Actinobacteria* | 0.14 | 0.14 | 0.14 | 0.15 |
|  | *Firmicutes* | 0.01 | 0.01 | 0.00 | 0.01 |
|  | *Ignavibacteriae* | 0.01 | 0.00 | 0.00 | 0.00 |
|  | Total | 2.49 c | 3.19 b | 3.56 a | 3.99 a |
| Aerobic chemoheterotrophy | *Acidobacteria* | 1.42 | 1.39 | 0.90 | 1.14 |
|  | *Proteobacteria* | 0.68 | 1.25 | 2.13 | 2.03 |
|  | *Bacteroidetes* | 0.19 | 0.39 | 0.39 | 0.67 |
|  | *Actinobacteria* | 0.14 | 0.14 | 0.14 | 0.15 |
|  | *Firmicutes* | 0.01 | 0.01 | 0.00 | 0.01 |
|  | *Ignavibacteriae* | 0.01 | 0.00 | 0.00 | 0.00 |
|  | Total | 2.44 c | 3.17 b | 3.56 a | 3.99 a |

Different letters are significantly different at *P* < 0.05 according to Duncan test.

**Supplementary TABLE 2 ǀ** Relationship between soil properties and the relative abundance of the main phyla and genera.

| Microbial community characteristics | | pH | | TN | | SOC | | EN | | AP | | C:N | |
| --- | --- | --- | --- | --- | --- | --- | --- | --- | --- | --- | --- | --- | --- |
|  |  | r | *P* | r | *P* | r | *P* | r | *P* | r | *P* | r | *P* |
| Phyla | *Proteobacteria* | 0.093 | 0.773 | 0.696**^*^** | 0.012 | 0.609**^*^** | 0.036 | 0.610**^*^** | 0.035 | 0.553 | 0.062 | -0.532 | 0.075 |
|  | *Actinobacteria* | 0.391 | 0.209 | -0.100 | 0.757 | 0.029 | 0.930 | -0.030 | 0.927 | 0.016 | 0.960 | 0.457 | 0.135 |
|  | *Acidobacteria* | 0.432 | 0.161 | 0.493 | 0.104 | 0.419 | 0.175 | 0.547 | 0.066 | 0.320 | 0.310 | -0.480 | 0.114 |
|  | *Chloroflexi* | 0.573 | 0.051 | -0.037 | 0.910 | 0.063 | 0.845 | 0.046 | 0.888 | -0.080 | 0.805 | 0.276 | 0.384 |
|  | *Planctomycetes* | -0.481 | 0.113 | -0.616**^*^** | 0.033 | -0.652**^*^** | 0.022 | -0.601**^*^** | 0.039 | -0.569 | 0.053 | 0.112 | 0.729 |
|  | *Bacteroidetes* | 0.081 | 0.803 | 0.628**^*^** | 0.029 | 0.556 | 0.060 | 0.503 | 0.096 | 0.450 | 0.142 | -0.463 | 0.130 |
|  | *Gemmatimonadetes* | -0.519 | 0.084 | -0.956**^**^** | 0.000 | -0.946**^**^** | 0.000 | -0.939**^**^** | 0.000 | -0.861**^**^** | 0.000 | 0.417 | 0.178 |
|  | *Nitrospirae* | 0.057 | 0.861 | 0.046 | 0.888 | 0.044 | 0.892 | 0.228 | 0.475 | 0.132 | 0.683 | -0.040 | 0.902 |
|  | *Firmicutes* | -0.679**^*^** | 0.015 | -0.674**^*^** | 0.016 | -0.640**^*^** | 0.025 | -0.682**^*^** | 0.014 | -0.432 | 0.161 | 0.387 | 0.214 |
|  | *Cyanobacteria* | -0.684**^*^** | 0.014 | -0.951**^**^** | 0.000 | -0.918**^**^** | 0.000 | -0.925**^**^** | 0.000 | -0.790**^**^** | 0.002 | 0.485 | 0.110 |
| Genera | *Gaiella* | -0.014 | 0.966 | -0.489 | 0.106 | -0.357 | 0.254 | -0.405 | 0.192 | -0.236 | 0.459 | 0.610**^*^** | 0.035 |
|  | *Microlunatus* | 0.240 | 0.453 | -0.410 | 0.185 | -0.375 | 0.229 | -0.416 | 0.179 | -0.609**^*^** | 0.036 | 0.272 | 0.392 |
|  | *Pseudarthrobacter* | 0.364 | 0.245 | 0.383 | 0.219 | 0.344 | 0.274 | 0.289 | 0.361 | 0.099 | 0.759 | -0.275 | 0.387 |
|  | *Blastococcus* | 0.058 | 0.858 | -0.598**^*^** | 0.040 | -0.568 | 0.054 | -0.616**^*^** | 0.033 | -0.650**^*^** | 0.022 | 0.332 | 0.291 |
|  | *Solirubrobacter* | 0.823**^**^** | 0.001 | 0.649**^*^** | 0.022 | 0.694**^*^** | 0.012 | 0.650**^*^** | 0.022 | 0.517 | 0.085 | -0.102 | 0.752 |
|  | *Rubrobacter* | 0.480 | 0.115 | -0.260 | 0.415 | -0.219 | 0.493 | -0.125 | 0.698 | -0.377 | 0.227 | 0.209 | 0.513 |
|  | *Nocardioides* | 0.467 | 0.126 | 0.843**^**^** | 0.001 | 0.833**^**^** | 0.001 | 0.762**^**^** | 0.004 | 0.720**^**^** | 0.008 | -0.353 | 0.260 |
|  | *Lapillicoccus* | 0.228 | 0.477 | -0.028 | 0.932 | 0.032 | 0.921 | -0.122 | 0.706 | -0.125 | 0.700 | 0.259 | 0.416 |
|  | *Marmoricola* | 0.503 | 0.095 | 0.429 | 0.164 | 0.454 | 0.138 | 0.387 | 0.213 | 0.306 | 0.334 | -0.075 | 0.817 |
|  | *Pseudonocardia* | 0.450 | 0.142 | 0.804**^**^** | 0.002 | 0.781**^**^** | 0.003 | 0.759**^**^** | 0.004 | 0.661**^*^** | 0.019 | -0.348 | 0.268 |
|  | *CL500-29_marine_group* | 0.818**^**^** | 0.001 | 0.716**^**^** | 0.009 | 0.706**^*^** | 0.010 | 0.744**^**^** | 0.005 | 0.480 | 0.114 | -0.348 | 0.267 |
|  | *Streptomyces* | -0.260 | 0.414 | -0.053 | 0.871 | -0.047 | 0.885 | -0.131 | 0.684 | 0.062 | 0.848 | 0.055 | 0.865 |
|  | *Mycobacterium* | 0.142 | 0.659 | -0.056 | 0.864 | -0.057 | 0.859 | -0.123 | 0.703 | -0.120 | 0.710 | 0.053 | 0.869 |
|  | *Crossiella* | -0.223 | 0.486 | -0.290 | 0.360 | -0.273 | 0.391 | -0.265 | 0.405 | -0.192 | 0.551 | 0.224 | 0.484 |
|  | *Oryzihumus* | -0.086 | 0.790 | -0.099 | 0.760 | -0.100 | 0.757 | -0.243 | 0.447 | -0.107 | 0.742 | 0.077 | 0.812 |
|  | *Lechevalieria* | 0.260 | 0.415 | -0.277 | 0.383 | -0.265 | 0.406 | -0.338 | 0.283 | -0.553 | 0.062 | 0.156 | 0.629 |
|  | *Kribbella* | 0.511 | 0.089 | 0.749**^**^** | 0.005 | 0.741**^**^** | 0.006 | 0.720**^**^** | 0.008 | 0.577**^*^** | 0.050 | -0.310 | 0.327 |
|  | *Patulibacter* | 0.435 | 0.158 | 0.643**^*^** | 0.024 | 0.661**^*^** | 0.019 | 0.506 | 0.093 | 0.550 | 0.064 | -0.153 | 0.634 |
|  | *Jatrophihabitans* | 0.181 | 0.572 | 0.120 | 0.710 | 0.201 | 0.531 | 0.155 | 0.631 | 0.269 | 0.398 | 0.273 | 0.390 |
|  | *Acidibacter* | 0.389 | 0.211 | 0.804**^**^** | 0.002 | 0.699**^*^** | 0.011 | 0.714**^**^** | 0.009 | 0.534 | 0.074 | -0.668**^*^** | 0.018 |
|  | *Anaeromyxobacter* | 0.336 | 0.286 | 0.325 | 0.302 | 0.452 | 0.140 | 0.273 | 0.391 | 0.454 | 0.138 | 0.280 | 0.378 |
|  | *Archangium* | -0.432 | 0.161 | -0.467 | 0.126 | -0.574 | 0.051 | -0.529 | 0.077 | -0.584^*^ | 0.046 | -0.145 | 0.654 |
|  | *Arenimonas* | 0.174 | 0.588 | 0.475 | 0.118 | 0.355 | 0.258 | 0.447 | 0.145 | 0.245 | 0.443 | -0.562 | 0.057 |
|  | *Azohydromonas* | 0.109 | 0.736 | 0.585**^*^** | 0.046 | 0.438 | 0.154 | 0.510 | 0.091 | 0.345 | 0.272 | -0.707**^*^** | 0.010 |
|  | *Bauldia* | 0.565 | 0.056 | 0.827**^**^** | 0.001 | 0.787**^**^** | 0.002 | 0.850**^**^** | 0.000 | 0.703**^*^** | 0.011 | -0.472 | 0.122 |
|  | *Bradyrhizobium* | 0.880**^**^** | 0.000 | 0.818**^**^** | 0.001 | 0.827**^**^** | 0.001 | 0.813**^**^** | 0.001 | 0.590**^*^** | 0.043 | -0.301 | 0.341 |
|  | *Defluviicoccus* | -0.282 | 0.374 | -0.439 | 0.153 | -0.555 | 0.061 | -0.515 | 0.087 | -0.515 | 0.086 | -0.210 | 0.513 |
|  | *Devosia* | 0.161 | 0.617 | 0.688**^*^** | 0.013 | 0.607**^*^** | 0.036 | 0.608**^*^** | 0.036 | 0.516 | 0.086 | -0.531 | 0.075 |
|  | *G55* | -0.153 | 0.634 | 0.091 | 0.779 | 0.094 | 0.772 | 0.154 | 0.633 | 0.252 | 0.430 | -0.023 | 0.942 |
|  | *Geobacter* | 0.575 | 0.050 | 0.679**^*^** | 0.015 | 0.656**^*^** | 0.020 | 0.612**^*^** | 0.035 | 0.501 | 0.097 | -0.374 | 0.232 |
|  | *H16* | -0.205 | 0.523 | -0.242 | 0.448 | -0.093 | 0.773 | -0.125 | 0.698 | 0.067 | 0.836 | 0.607^*^ | 0.037 |
|  | *Haliangium* | 0.440 | 0.152 | 0.854**^**^** | 0.000 | 0.893**^**^** | 0.000 | 0.931**^**^** | 0.000 | 0.885**^**^** | 0.000 | -0.189 | 0.556 |
|  | *Labrys* | 0.779^**^ | 0.003 | 0.743**^**^** | 0.006 | 0.739**^**^** | 0.006 | 0.715**^**^** | 0.009 | 0.541 | 0.069 | -0.336 | 0.286 |
|  | *Lysobacter* | 0.197 | 0.539 | 0.745**^**^** | 0.005 | 0.682**^*^** | 0.015 | 0.645**^*^** | 0.024 | 0.598**^*^** | 0.040 | -0.481 | 0.114 |
|  | *Massilia* | -0.304 | 0.336 | -0.394 | 0.206 | -0.382 | 0.220 | -0.409 | 0.187 | -0.312 | 0.323 | 0.188 | 0.559 |
|  | *Mesorhizobium* | 0.573 | 0.052 | 0.923**^**^** | 0.000 | 0.866**^**^** | 0.000 | 0.867**^**^** | 0.000 | 0.709**^**^** | 0.010 | -0.559 | 0.059 |
|  | *Methylorosula* | -0.280 | 0.379 | -0.849**^**^** | 0.000 | -0.881**^**^** | 0.000 | -0.876**^**^** | 0.000 | -0.920**^**^** | 0.000 | 0.201 | 0.531 |
|  | *Microvirga* | -0.394 | 0.205 | -0.344 | 0.274 | -0.346 | 0.271 | -0.360 | 0.251 | -0.234 | 0.465 | 0.124 | 0.700 |
|  | *Nordella* | 0.355 | 0.258 | 0.122 | 0.705 | 0.143 | 0.658 | 0.028 | 0.932 | -0.106 | 0.744 | 0.027 | 0.933 |
|  | *Noviherbaspirillum* | -0.415 | 0.179 | -0.917**^**^** | 0.000 | -0.949**^**^** | 0.000 | -0.928**^**^** | 0.000 | -0.971**^**^** | 0.000 | 0.240 | 0.452 |
|  | *Pedomicrobium* | 0.801**^**^** | 0.002 | 0.860**^**^** | 0.000 | 0.898**^**^** | 0.000 | 0.895**^**^** | 0.000 | 0.732**^**^** | 0.007 | -0.231 | 0.471 |
|  | *Phenylobacterium* | 0.034 | 0.915 | 0.456 | 0.136 | 0.368 | 0.239 | 0.377 | 0.228 | 0.310 | 0.328 | -0.418 | 0.177 |
|  | *Piscinibacter* | 0.163 | 0.613 | 0.598**^*^** | 0.040 | 0.474 | 0.120 | 0.495 | 0.102 | 0.392 | 0.207 | -0.626^*^ | 0.029 |
|  | *Pseudomonas* | -0.031 | 0.924 | 0.166 | 0.607 | 0.131 | 0.685 | 0.188 | 0.558 | 0.148 | 0.647 | -0.205 | 0.523 |
|  | *Reyranella* | 0.542 | 0.069 | 0.901**^**^** | 0.000 | 0.848**^**^** | 0.000 | 0.925**^**^** | 0.000 | 0.765**^**^** | 0.004 | -0.515 | 0.087 |
|  | *Rhizomicrobium* | -0.534 | 0.074 | -0.207 | 0.519 | -0.135 | 0.677 | -0.127 | 0.694 | 0.193 | 0.547 | 0.360 | 0.250 |
|  | *Rhodoplanes* | 0.806**^**^** | 0.002 | 0.767**^**^** | 0.004 | 0.790**^**^** | 0.002 | 0.790**^**^** | 0.002 | 0.617**^*^** | 0.033 | -0.233 | 0.466 |
|  | *Skermanella* | -0.496 | 0.101 | 0.071 | 0.826 | 0.007 | 0.982 | 0.008 | 0.980 | 0.045 | 0.889 | -0.196 | 0.542 |
|  | *Sphingomonas* | -0.268 | 0.399 | -0.334 | 0.289 | -0.367 | 0.241 | -0.428 | 0.165 | -0.352 | 0.262 | 0.009 | 0.977 |
|  | *Steroidobacter* | 0.241 | 0.450 | 0.809**^**^** | 0.001 | 0.720**^**^** | 0.008 | 0.705**^*^** | 0.010 | 0.608**^*^** | 0.036 | -0.602**^*^** | 0.038 |
|  | *Variibacter* | 0.559 | 0.059 | 0.795**^**^** | 0.002 | 0.748**^**^** | 0.005 | 0.703**^*^** | 0.011 | 0.544 | 0.067 | -0.450 | 0.142 |
|  | *RB41* | -0.208 | 0.517 | -0.650**^*^** | 0.022 | -0.686**^*^** | 0.014 | -0.608**^*^** | 0.036 | -0.776**^**^** | 0.003 | 0.137 | 0.671 |
|  | *Candidatus_Koribacter* | -0.673**^*^** | 0.016 | -0.368 | 0.239 | -0.446 | 0.146 | -0.550 | 0.064 | -0.378 | 0.225 | -0.069 | 0.832 |
|  | *Candidatus_Solibacter* | -0.470 | 0.123 | -0.846**^**^** | 0.001 | -0.839**^**^** | 0.001 | -0.790**^**^** | 0.002 | -0.719**^**^** | 0.008 | 0.323 | 0.305 |
|  | *Bryobacter* | -0.592**^*^** | 0.043 | -0.843**^**^** | 0.001 | -0.917**^**^** | 0.000 | -0.839**^**^** | 0.001 | -0.819**^**^** | 0.001 | 0.058 | 0.857 |
|  | *11-24* | -0.367 | 0.241 | -0.643**^*^** | 0.024 | -.695**^*^** | 0.012 | -0.539 | 0.071 | -0.629**^*^** | 0.028 | 0.044 | 0.891 |
|  | *Ferruginibacter* | 0.099 | 0.759 | 0.591^*^ | 0.043 | 0.503 | 0.096 | 0.478 | 0.116 | 0.390 | 0.210 | -0.503 | 0.096 |
|  | *Flavisolibacter* | -0.437 | 0.156 | -0.736^**^ | 0.006 | -0.759^**^ | 0.004 | -0.834^**^ | 0.001 | -0.791^**^ | 0.002 | 0.229 | 0.473 |
|  | *Segetibacter* | -0.331 | 0.294 | -0.821^**^ | 0.001 | -0.810^**^ | 0.001 | -0.864^**^ | 0.000 | -0.807^**^ | 0.002 | 0.361 | 0.249 |
|  | *Chitinophaga* | -0.298 | 0.346 | -0.206 | 0.521 | -0.192 | 0.549 | -0.237 | 0.459 | -0.119 | 0.713 | 0.125 | 0.698 |
|  | *Parafilimonas* | -0.750^**^ | 0.005 | -0.310 | 0.326 | -0.377 | 0.227 | -0.370 | 0.237 | -0.158 | 0.623 | -0.056 | 0.863 |
|  | *Pirellula* | -0.496 | 0.101 | -0.449 | 0.143 | -0.473 | 0.120 | -0.398 | 0.200 | -0.423 | 0.170 | 0.092 | 0.775 |
|  | *Gemmata* | 0.118 | 0.714 | -0.034 | 0.916 | -0.115 | 0.723 | -0.138 | 0.668 | -0.169 | 0.600 | -0.272 | 0.392 |
|  | *Pir4_lineage* | -0.321 | 0.309 | -0.367 | 0.241 | -0.368 | 0.239 | -0.407 | 0.190 | -0.279 | 0.380 | 0.104 | 0.747 |
|  | *Singulisphaera* | -0.296 | 0.350 | -0.392 | 0.208 | -0.385 | 0.217 | -0.361 | 0.249 | -0.346 | 0.271 | 0.182 | 0.572 |
|  | *Zavarzinella* | 0.050 | 0.878 | -0.497 | 0.100 | -0.596^*^ | 0.041 | -0.496 | 0.101 | -0.693^*^ | 0.013 | -0.180 | 0.575 |
|  | *Planctomyces* | 0.484 | 0.111 | 0.913^**^ | 0.000 | 0.845^**^ | 0.001 | 0.855^**^ | 0.000 | 0.734^**^ | 0.007 | -0.597^*^ | 0.041 |
|  | *Bacillus* | -0.660^*^ | 0.020 | -0.694^*^ | 0.012 | -0.648^*^ | 0.023 | -0.681^*^ | 0.015 | -0.444 | 0.148 | 0.436 | 0.157 |
|  | *Paenibacillus* | -0.535 | 0.073 | -0.611^*^ | 0.035 | -0.599^*^ | 0.039 | -0.587^*^ | 0.045 | -0.445 | 0.147 | 0.271 | 0.394 |
|  | *Nitrospira* | 0.046 | 0.887 | 0.070 | 0.828 | 0.044 | 0.891 | 0.231 | 0.470 | 0.132 | 0.683 | -0.132 | 0.683 |
|  | *Roseiflexus* | 0.497 | 0.100 | -0.217 | 0.498 | -0.222 | 0.487 | -0.090 | 0.780 | -0.455 | 0.138 | -0.002 | 0.994 |
|  | *Gemmatimonas* | -0.461 | 0.131 | -0.801^**^ | 0.002 | -0.850^**^ | 0.000 | -0.821^**^ | 0.001 | -0.813^**^ | 0.001 | 0.167 | 0.605 |
|  | *Chthonomonas* | -0.483 | 0.112 | -0.688^*^ | 0.013 | -0.780^**^ | 0.003 | -0.670^*^ | 0.017 | -0.700^*^ | 0.011 | -0.032 | 0.922 |
|  | *Opitutus* | 0.114 | 0.724 | 0.676^*^ | 0.016 | 0.582^*^ | 0.047 | 0.586^*^ | 0.045 | 0.531 | 0.076 | -0.574 | 0.051 |

*: *P* < 0.05; **: *P* < 0.01.

TN: total nitrogen; SOC: soil organic carbon; EN: exchangeable nitrogen; AP: available phosphorus.
